# Design of a randomized controlled trial of extended-release naltrexone versus daily buprenorphine-naloxone for opioid dependence in Norway (NTX-SBX)

**DOI:** 10.1186/s40360-016-0061-1

**Published:** 2016-04-28

**Authors:** Nikolaj Kunøe, Arild Opheim, Kristin Klemmetsby Solli, Zhanna Gaulen, Kamni Sharma-Haase, Zill-e-Huma Latif, Lars Tanum

**Affiliations:** Norwegian Centre for Addiction Research, University of Oslo, PO Box 1039, Blindern, N-0315 Oslo, Norway; Department of Research and Development, Mental Health Services, Akershus University Hospital, Lørenskog, Norway; Department of Addiction Medicine, Haukeland University Hospital, Bergen, Norway; The University of Bergen, Bergen, Norway; Vestfold Hospital Trust, Tønsberg, Norway

**Keywords:** Substance abuse, Opioid addiction, Naltrexone, Extended-release, Study design, Methodology

## Abstract

**Background:**

Current guidelines for opioid dependence recommend daily maintenance of physical dependence with methadone or buprenorphine, and discourage abstinence due to the high risk of relapse and overdose. Extended-release formulations of the opioid antagonist naltrexone (XR-NTX) block heroin and other opioid agonists competitively for around 4 weeks per administration. XR-NTX thus enables opioid users to experience abstinence from opioid agonists with greatly reduced risk of overdose compared to medication-free abstinence. While XR-NTX has shown promise compared to placebo and daily naltrexone tablets, there is limited information on long-term safety and its performance compared to daily maintenance treatment.

**Methods/Design:**

In this five-hospital RCT with long-term follow-up, we aim to recruit *n* = 180 patients in treatment for opioid dependence and allocate them in an open, randomized manner (1:1) to receive either 4-week XR-NTX or daily buprenorphine-naloxone (BP-NLX) for the duration of 12 weeks. Allocation is open-label due to the risk of overdose during attempts to self-unmask allocation using heroin. Urine drug tests are scheduled every week with follow-up visits & assessment every 4 weeks. Primary outcomes are abstinence from illicit opioids in urine drug tests and self-report, as well as retention in treatment. Secondary outcomes include other substance use, injecting behavior, drug craving, mental health, quality of life, treatment satisfaction, abstinence motivation, opioid agonist effect rating, insomnia, and pain. Observation is continued for another 36 weeks in order to assess longer-term safety, adherence and effectiveness. The study is an investigator-initiated trial, funded by public grants and approved by an Independent Ethical Committee (the Regional Ethical Committee for Research South-East B # 2011/1320) and the Norwegian Medicines Agency.

**Discussion:**

Despite minor implementation problems, the protocol appears sufficiently robust to generate results of high interest to patients, clinicians and policy makers.

**Trial registration:**

Clinicaltrials.gov # NCT01717963, first registered: Oct 28, 2012. Protocol version # 3C, June 12th 2012.

## Background

Death from opioid overdose is the leading cause of death among drug users, and has more than doubled in the US over the last 15 years [1, 2] in what is often characterized as an ‘overdose epidemic.’ A main mechanism is the development of addiction to opioid agonist drugs like heroin and/or illicit diversion of prescribed opioid medications [3]. A chronically relapsing disorder [4], addiction to illicit opioids often includes criminal activity, poly-drug use and blood-borne infections such as HIV [5].

Due to high risk of relapse and overdose associated with abstinence, the preferred treatment for opioid addiction is substitution of the illicit opioid with a prescribed opioid medication like the full opioid agonist methadone [6] or partial opioid agonist buprenorphine [5]. This agonist replacement therapy (ART) is generally effective at reducing illicit opioid use, overdose mortality [7], as well as important drug-related outcomes such as criminal activity [8] and injection-related disease [9].

As ART presents a risk of illicit diversion and abuse of prescribed methadone or buprenorphine [10], ART providers will often attempt to minimize this risk by utilizing control measures such as supervised medication intake, urine drug screens, limits on take-home dosing and–travel outside the catchment area [5, 11]. A disadvantage of these control measures is their tendency to reduce patients’ sense of personal freedom, potentially undermining their motivation to remain in ART [5]. As methadone, a full opioid agonist, is considered to have a high potential for abuse and overdose, the partial opioid agonist buprenorphine is sometimes preferred. The abuse risk of buprenorphine can be further reduced when combined with a component of the short-acting opioid antagonist naloxone; this combination product tends to not cause withdrawal when ingested correctly (sublingually), but is likely to cause some withdrawal symptoms if the medication is injected [12].

Despite the dilemmas inherent in treating opioid users with opioid agonists, current guidelines recommend ART due to its ability to retain patients in treatment while greatly reducing illicit opioid use and overdose risk [5]. This in contrast to the other main treatment principle, medication-free abstinence, which is generally not recommended due to the elevated risk of overdose following a period of abstinence from opioid agonists. Despite the risks, treatment based on medication-free abstinence is available in most countries, e.g. as long-term residential treatment or therapeutic communities with outpatient follow-up.

A main concern for opioid addiction treatment is its limited uptake in the user population: Only about 50 % of the patient base are estimated to be in treatment at any one time [5, 13]. The potential human and social benefit of increasing the number of opioid users in treatment is the main incentive to exploring novel treatment approaches.

One recent innovation is sustained release formulations of the opioid antagonist naltrexone [14–16] (SRX). Following detoxification from all opioid agonists (e.g. heroin, morphine, methadone, buprenorphine), naltrexone medication will support the person in avoiding relapse by rendering attempts at relapse futile: as naltrexone competitively blocks the action of heroin and other opioid agonists, the drug euphoria (or ‘high’) as well as sedative effects should effectively be inaccessible to the patient. The only version of SRX currently approved by the FDA, extended release naltrexone (XR-NTX), is administered intramuscularly in the gluteus where it releases naltrexone at therapeutic levels for the duration of 4–5 weeks per injection. XR-NTX appears superior to placebo in both laboratory and clinical settings [17–19]; other sustained release naltrexone (SRX) formulations have shown promising results compared to daily oral naltrexone [20, 21] and to usual-treatment controls [22, 23].

Given the success of ART in clinical practice, a valid policy question is the need to implement another treatment like extended-release naltrexone for the target disorder, opioid dependence. This is best addressed by a randomized controlled trial of long-acting naltrexone with the currently most similar ART medication on offer, daily ART with buprenorphine-naloxone.

In addition to the advantages offered by a randomized comparison, most observers emphasize the need for observation beyond the 8 to 24 weeks often reserved for RCTs [3, 4, 24]. With XR-NTX, only a handful of studies have so far investigated long-term outcomes [25–27]. Long-term observation will also improve the accuracy estimates of occurrence of adverse events. Oral naltrexone tablets has been available since the 1980s, but lack of adherence undermines clinical efficacy [28] and creates uncertainty about the validity of safety data.

Although naltrexone has shown the ability to reduce craving for several substances of abuse e.g. alcohol [29, 30] and heroin [31], there is still clinical interest in whether poly-drug using patients will increase non-opioid substance use during XR-NTX. Other long-term consequences of opioid antagonism are also subject to debate, mainly based on preclinical and/or case data: Some suggest naltrexone could reverse receptor-level changes attributed to chronic opioid agonist intake [32]; others think it plausible naltrexone could increase users’ vulnerability to overdose upon discontinuation of the medication [33] due to chronic antagonism stimulating receptor growth and sensitivity.

The present protocol describes a study of adult opioid users randomized to receive either 4-week intramuscular naltrexone (XR-NTX) or daily buprenorphine-naloxone (BP-NLX) in a clinical setting. The purpose of the study is to explore recovery–and medication-related outcomes of similarly motivated patients randomized to either treatment. For ethical reasons, patients will not be discontinued from XR-NTX but permitted to receive XR-NTX for up to 48 Weeks total, providing an opportunity to observe longer-term effects of chronic medication with XR-NTX.

## Methods/Design

### Study design

This is a 12-week open-label randomized-controlled trial comparing 4-week intramuscular injections of extended release naltrexone (XR-NTX) to a currently recommended treatment for opioid addiction, daily buprenorphine-naloxone (BP-NLX). As this is he first RCT comparing these two medications, we consider the scientific approach to be exploratory.

For ethical reasons (see below) study medication (XR-NTX) will not be terminated upon RCT completion, but will be available for an additional 36 weeks (48 weeks total observation time). This will produce long-term data clinical safety and effectiveness of chronic naltrexone treatment. Registry data collection of mortality, morbidity, criminal justice and prescription records will take place before, during and after the prospective data observation in order to provide additional information on the volunteers and long-term effects of XR-NTX medication.

### Research questions and hypotheses

The main research question is whether 4-week XR-NTX will be more effective than–or equally effective to a current standard treatment–daily BP-NLX–on primary and secondary outcomes during or at the end of the 12-Week study.

As an exploratory study with both non-superiority and superiority as plausible scenarios [34] there are two sets of hypotheses for this RCT:

XR-NTX will influence primary and secondary outcomes to be either:

- statistically superior to assignment to the current standard treatment, ART with daily BP-NLX or
- statistically non-superior to ART with daily BP-NLX.

### Research ethics

The Regional Ethical Board for Medical Research Ethics, committee South East B approved the study protocol in 2011 (#2011/1320). Any significant modifications to the protocol will be forwarded to the committee for approval.

The ethical obligation of the Helsinki Declaration to minimize harm to research participants, specifically the risk of overdose, has influenced two main design characteristics:

- To administrate medications openly (open-label) rather than use a masked, placebo-controlled design. While a placebo design increases internal validity and the precision of efficacy estimates, generalizability and estimates of clinical effectiveness may be reduced. In particular, a placebo design with this population risks ‘self-unmasking’ by use of illicit opioids [35] reducing the potential benefit of a masking procedure and increasing risk of illicit opioid use by participants, enhancing their vulnerability to overdose and relapse during or after study participation.
- Because transferring patients to standard treatment would either re-instate physical dependence on prescribed opioids like methadone in ART or risk relapse and overdose in medication-free abstinence, we consider the best solution to offer continuation of XR-NTX medication to participants post completion of the 12-Week phase as the alternative. This will also enable the collection of much needed long-term data on XR-NTX. BP-NLX patients who wish to continue on their medication will be offered to do so as part of the national ART program.

### Participants & setting

Patients have been recruited during the period from November 2012 to July 2015 by trained personnel based at five research hospitals in urban centers in Norway: Oslo University Hospital, Akershus University Hospital, Haukeland University Hospital, Stavanger University Hospital, and Vestfold Hospital Trust. An agreement is in place between the study management and the Directorate of Norwegian Correctional Service for study sites to recruit opioid users who are completing criminal sentencing in criminal justice facilities.

Opioid dependent (DSM-IV) adults (18+) without serious mental or somatic disease are eligible to participate: study personnel screen patients for acute or chronic suicidality or psychotic disorders using the MINI 6.0 interview [36], while a physician examines patients for serious somatic disease such as acute hepatic failure (Child-Pugh level 3+) or AIDS-indicator disease. Eligible patients can also be screened in an outpatient setting before entering detoxification in a protected environment (inpatient or closely monitored outpatient). All patients must be formally enrolled in Norway’s national ART program at one of the study sites in order to be eligible for randomization. This ensures personal follow-up by a counselor as part of standard outpatient treatment, and rapid access to other ART program resources (e.g. prescribed methadone) for patients who drop out of the study treatments. As the effects of XR-NTX on fetal development are not yet known, female participants are screened for pregnancy during inclusion and assisted in finding effective contraception for the duration of the study. Patients treated with opioid-based analgesics for chronic pain conditions will only be eligible if successfully transferred to a non-opioid replacement (e.g. benzodiazepines).

### Recruitment procedure

Participants are recruited via written information on brochures, webpages, and posters at site clinics, and via personal contact with their counselor or physician. After receiving verbal and written information about the study from study staff, patients are screened for inclusion/exclusion criteria and sign the written informed consent. The consent form is based on the template of the Regional Ethical Committee for Medical Research downloaded from http://helseforskning.etikkom.no/ikbViewer/Content/253516/TemplateClinicalTrial%20revised%2020120209.doc Separate consent forms will be utilized in the event that ancillary studies are started that are outside the scope of the present consent.

#### Pre-randomization tapering of opioid agonists

As naltrexone may induce withdrawal symptoms by displacing any opioid agonists at opioid receptor sites, they are assisted in tapering and detoxification in order to facilitate the transition with as low a risk as possible. Detoxification and induction on to study medication is conducted in a controlled environment, usually an on-site inpatient detoxification unit. Only patients unwilling or assessed as unable to undergo inpatient detoxification, an outpatient detoxification with daily staff contact can be arranged with daily or bi-daily urine drug screening.

Buprenorphine is the recommended medication for alleviating opioid withdrawal symptoms during detoxification in Norway, and is administered to all participants at the discretion of the on-site treating physician. A higher dose is typically administered to ART patients habituated to a daily buprenorphine dose (16 mg/buprenorphine is the standard target dose in ART) tapered for a standard 2 mg/day, while a lower dose (2–4 mg buprenorphine/day) is typically administered to illicit heroin users. Any regular methadone users are required to taper down and discontinue methadone for a minimum of 72 h before randomization. Once participants are stable in the 0–4 mg buprenorphine/day range, they are considered ready for random allocation (1:1) to either standard treatment with buprenorphine-naloxone (target dosage 16 mg/day, range 4–24 mg) or extended-release naltrexone (XR-NTX) 380 mg every 28 days, for the upcoming 12 weeks/84 days.

### Randomization

Allocation is conducted by non-study personnel on a per-patient basis using a permuted block algorithm accessible via a computer program with full-entry tracking. Participants are randomly allocated with a 1:1 ratio to either XR-NTX or BP-NLX independent of site, setting (clinic or criminal justice) or gender. When a study staff member contacts allocation staff for inclusion and randomization, inclusion criteria are verified before the patient is formally included into the database and allocated to one of the two medication groups. Group allocation is communicated openly to study staff via telephone, who are then free to communicate the results of the allocation procedure to patient and clinical staff in an open-label manner.

### Study medications

Before discharge from a controlled environment, buprenorphine-naloxone patients are inducted on to a flexible dose of BP-NLX: Range 4–24 mg/day of buprenorphine, target 16 mg/day, with a 1/4 naloxone component (range 1–6 mg; 4 mg in the target dose). The approach is based on Norway’s national ART guideline on buprenorphine pharmacotherapy for opioid dependence, which is based on an evaluation of the scientific literature. For the BP-NLX group, medication is provided courtesy of existing healthcare services for opioid users in Norway.

For patients allocated to receive extended-release naltrexone (XR-NTX), tapering is continued with a flexible, standard 2 mg/day regimen followed by a minimum of 72 h without opioid agonists in order to prevent inducing withdrawal symptoms. A naloxone challenge (0.2–0.4 mg) is administered prior to the first XR-NTX (380 mg) dose in order to confirm the absence of opioid agonist. Following induction onto either study drug (usually 3–7 days), patients are discharged from detoxification and are requested to attend regular ART program counseling and present weekly urine drug tests for the next 12 weeks. As XR-NTX is currently not available for purchase in Europe, study management requested the manufacturer to supply it free of charge for this study without additional funding and without conditions for manufacturer influence over study implementation, analysis or dissemination. The XR-NTX manufacturer (Alkermes, Inc) complied with this request.

After the completion of the 12-Week follow-up, participants are asked whether they wish to continue for the long-term observation phase of the study (48 Weeks total observation time).

BP-NLX patients who wish to continue on their medication will be free to do so as part of the national ART program, but conclude regular participation in the study. BP-NLX patients who wish to transfer to XR-NTX will undergo a washout period with detoxification and naloxone test similar to procedures described for the XR-NTX induction (above).

### Outpatient follow-up

**Fig. 1:**
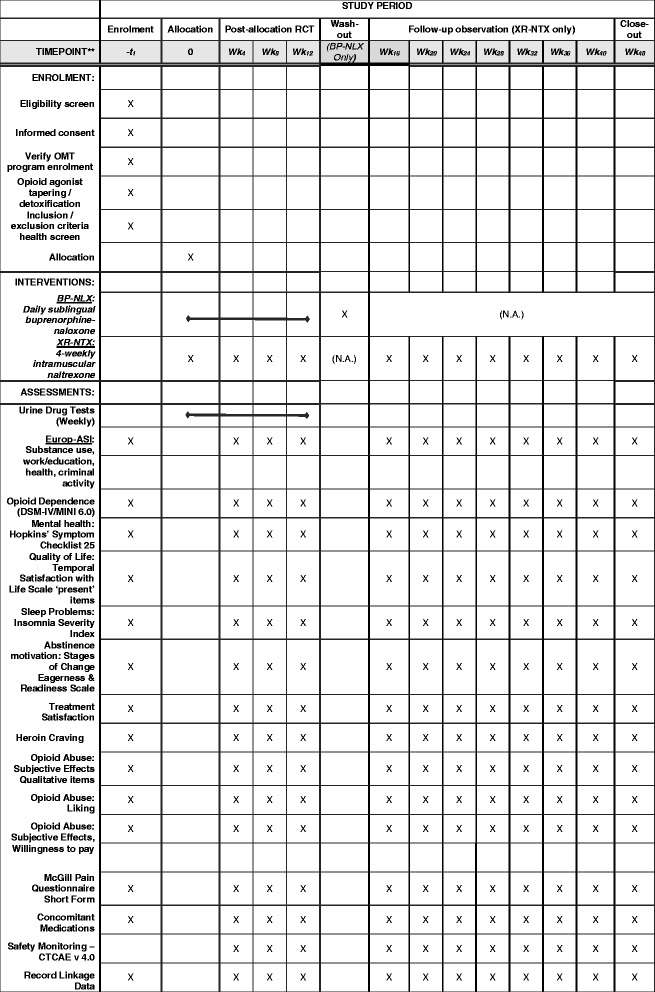
SPIRIT Flowchart of Study Events for the study’Optimal Prevention of Overdose Deaths and Opioid Relapse Following Discharge: A Multi-Center RCT of Naltrexone versus Buprenorphine in Norway’

Weekly urine drug tests (UDTs) are administered on site, at the local laboratory or at the GP’s office. A scratch card incentive is offered for each UDT (a maximum of 12) in order to increase UDT attendance. Every 4 weeks, participants have scheduled follow-up with data collection on all outcomes of interest (see Fig. 1). This coincides with re-administration of XR-NTX for patients allocated to this group and could coincide with one of four standard weekly counseling sessions offered in the on-site ART clinic. All 4-week assessments (except UDTs) are continued for patients who complete the 12-week RCT and elect to participate in the 36-week long-term observation phase. Adherence is monitored through self-report and can be verified by consulting each patient’s electronic journal on the site clinic, where each dose collected of either BP-NLX or XR-NTX is registered.

### Discontinuation

Discontinuation can occur on the participants’ request and will be considered by the investigator should the participant develop conditions contrary to inclusion criteria; e.g. acute or repeated psychotic episodes or suicide attempts, life-threatening or debilitating somatic conditions not compatible with participation (e.g. pharmacological incompatibility, preventing outpatient follow-up), assessed by a physician to have need for opioid-based analgesics with no feasible non-opioid replacements, pregnancy, or failure to adhere to study medication regimens. For BP-NLX this study adheres to clinical guidelines for ART where a flexible regimen is used to determine the dosage based on feedback on withdrawal and craving symptoms; a failure to attend BP-NLX administration for two consecutive days or more is considered non-adherence. For XR-NTX patients, adherence conditions are violated if re-dosing occurs more than 4 days past the standard 28-day re-dosing interval. In both groups, site investigators may make exceptions for credible force majeure scenarios. As overdose risk is known to increase upon discontinuation of addiction treatment, investigators have been asked to facilitate transfer to regular ART for patients who wish to discontinue either study treatment.

### Data management

Study personnel are trained to administer the structured Europ-ASI interview (see below) as well as the other instruments and CRFs used in the study. All data are entered into a GCP-compliant database with complete entry tracking and double entry/verification. Following completion of the study, data will be de-identified and stored for an additional 15 years accessible to the sponsor in compliance with Norwegian and European regulations. Due to the sensitivity of data and the protection of privacy of individual participants the dataset, there are no plans to make data available in a repository or public database.

#### Regulatory approvals, data–and safety monitoring

The Common Terminology Criteria for Adverse Events (CTCAE) version 4 is used to classify adverse events at follow-up. Any event requiring medical intervention is registered (CTCAE level 2 or above). Medication-related Severe Adverse Events (SAEs) and Sudden Unexpected Severe Adverse Reactions (SUSARS) are reported to the Norwegian Medicines Agency (European Medicines Agency) and to the study drug manufacturers (Alkermes, Inc. and Reckitt-Benckiser, Inc.) within two working days. No interim analyses are planned, but the Sponsor (the University of Oslo, Norway) as well as regulatory authorities may stop recruitment and further medication with study medication in the case of an emergency, e.g. an unusual accumulation of SAEs or SUSARs attributable to study medication.

As all participants are enrolled in a local ART clinic, ART services are assigned the legal responsibility for monitoring of everyday safety of the patient with the option of discontinuing patients from ART if necessary (and thus from study participation). In addition, a local data and safety monitoring committee consisting of site investigator, site coordinator, and a representative of the clinical monitoring service are responsible for assessing data quality and safety at each study site. National management consisting of LT, and NK, conducts additional supervision, provide training and guidance and assess compliance with the study protocol of all serious adverse events. As this is an investigator-initiated trial, there are no competing interests and the sponsor, the University of Oslo, Norway, is represented by the National PI (LT).

All relevant study personnel are trained in ICH-GCP and CTCAE event registration. All participants are insured against SAEs attributable to study drug (XR-NTX) occurring during or after participation via the Sponsor’s membership in the Norwegian Drug Liability Association.

The regional monitoring authorities at the Oslo University Hospital and at Haukeland University Hospital are utilized as GCP monitoring services for this study. Site monitoring will occur after the first patient at each site, after the initial *n* = 10 at each site, and yearly until last patient has completed or discontinued participation and final monitoring of *n* = 10 random client report files (CRFs) at each site. The Norwegian Medicines Agency may conduct audits of the trial at any time without notifying the sponsor or investigators.

#### Outcomes & instruments

Urine Drug Test (UDT) data will be analyzed in accordance with recent Cochrane specifications as either a) proportion of total number of UDTs without illicit opioids or b) number of UDTs negative for illicit opioids received from each patient in each group during the 12-week randomized period [37].

Retention in treatment is defined in two ways: Number of days (of maximum 84 days) receiving effective pharmacological treatment, and number of patients completing the study at Week 12.

At baseline and every 4 weeks, patients undergo a structured interview and a selection of patient-reported outcomes: The European version of the Addiction Severity Index (the Europ-ASI) [38, 39] comprises drug and alcohol use, physical and mental health, work, education, social relations, and criminal behavior. The Europ-ASI is used for the second main drug use outcome measure, the number of days of abstinence from illicit opioid use in the 28 days preceding each interview for a total (maximum) of 84 days. To increase accuracy of responses relative to the Europ-ASI standard, timeline follow-back is used: a technique of backtracking and questioning about use and abstinence to obtain as accurate an estimate as possible of total number of days’ use of a specific substance in the 28-day follow-up period [40].

In addition to the primary outcomes, we explore secondary outcomes of importance to recovery: Number of patients fulfilling MINI [36] (DSM-IV) criteria for dependence on illicit opioids (exempting the 12-month criterion). The Addiction Severity Index administered every 4th week also records information on the use/abuse of alcohol, amphetamines, cannabis, sedatives (e.g. benzodiazepines), as well as other illicit substances (e.g. LSD, MDMA, GHB), and poly-drug use; days of injecting drug use; any overdoses (non-lethal) and hospitalizations in somatic or psychiatric health care; workdays and days engaged in illegal activities; number of days with conflicts in close relations, days physical and mental illness.

Craving for heroin is assessed using a 0–10 Visual analogue scale (VAS) on two questions: ‘I need heroin’ where ‘0’ signifies ‘Strongly disagree’ and 10 ‘Strongly agree’; and ‘How much have you been bothered by thinking about opioids or their use in the past 4 weeks?’ with 0 indicating ‘not at all’ and 100 ‘constantly’.

Mental health is assessed using the 25-item Hopkins Symptom Checklist [41], life satisfaction using the Temporal Satisfaction With Life Scale ‘present’ items [42]. Treatment satisfaction was assessed using a visual analogue scales of 0–10 on the questions ‘How satisfied/dissatisfied are you with having had/not having received XR-NTX in the previous 4 weeks?’ and ‘To what extent would you recommend XR-NTX to a friend who was in the same situation you were in when entering this study?’. Based on previous studies [17, 43] the frequency and euphoria (‘high’) from any illicit opioid use was investigated using visual analogue scales on a qualitative description of any effects, the similarity of the experience to un-blocked drug euphoria (‘high’), what they would be willing to pay for a similar experience had they been in active drug use. In addition, motivation for abstinence using the SOCRATES-8 days [44], and pain assessment using the McGill Pain Questionnaire Short Form [45]. Norwegian language versions are used for all instruments.

Priority will be given to collection of primary outcomes for participants who are assessed to be unable (e.g. due to intoxication) to complete follow-up on one or more visits.

#### Registry data follow-up

The informed consent and Regional Ethical Board approval permits collection of registry linkage data based on the participants’ unique Personal Identity Number given to every permanent resident or employee in Norway upon birth or first residential/employment visa. The PIN is necessary for personal identification to public authorities and to open a Norwegian bank account. The PIN is also used in many registries and makes it possible to link information on individuals across registries [46]. Ethical Board approval has been granted to collect registry data for the study period and up to 1 year before, during and after participation in order to: a) compare data and events occurrence within the group and compare with other groups of interest and b) obtain data on patients who discontinue XR-NTX treatment. Data from the following registries will be included: The Cause of Death Registry, the Norwegian Prescription Database (NorPD), the Police Registry, the Norwegian Prison Registry and the Norwegian Patient Registry (NPR).

### Statistical analyses

Statistical analyses on the randomized part of the study will be conducted by a non-study statistician on a final RCT data set in which the two study medications is masked as ‘Rx A’ and ‘Rx B’; any obvious characteristics of the two comparison medications will also be censored before data are made available for analysis. For between- group comparisons and to control for the effect of baseline characteristics, we will use a General Linear Mixed Models (GLMM) approach containing factors for treatment group, sex, and sex-by- treatment interaction, and with age, duration of opioid dependence, and duration of last pre-study inpatient detoxification as covariates. For outcomes violating the normality assumption, a General Alinear Mixed Models (GAMM) approach will be used. Regular statistics (e.g. ANOVA, linear regression) may be utilized if the arguments for using GLMM or GAMM over legacy analysis types are considered to provide limited benefit. Retention will be assessed with Kaplan-Meier curves and a log-rank test. Adverse events will be compared using Fisher’s exact test. The primary endpoints are tested with a two-sided α = 0.05. Where relevant, *p* values will be adjusted for multiplicity using the Bonferroni-Holm method to preserve family-wise type 1 error at 0.05. Once the statistician concludes data analysis of the RCT phase, study management will be able to access the final dataset.

#### Determination of sample size

In compliance with current recommendations for non-superiority trials [34], a minimum sample size has been estimated for two scenarios:

The superiority scenario assumes that XR-NTX participants will have opioid-negative samples on a mean of 7 out of the total 12 (7/12 or 0.58) samples, while participants receiving buprenorphine-naloxone will display a mean of 4 opioid-negative samples (4/12 or 0.33). With a 95 % significance level (*p* < 0.05) and a standard deviation of 3 in both medication groups, and power (beta) set to 90 %, a sample size of 17 patients/medication arm or *n* = 34 total is estimated to be sufficient for analysis. The estimated frequency of opioid use in each group is based on data from Norwegian patients receiving sustained release naltrexone [22] or on the frequency of illicit opioid use during buprenorphine maintenance in the Norwegian national ART program.

In the non-superiority scenario, using a beta of 90 %, we assume that both groups will retain 70 % of their participants at the end of Week 12 and set 20 % as the non-superiority margin; this yields a minimum sample size of *n* = 58 in each group/*n* = 116 total.

Based on the sample size calculations and the risk of dropout of a significant proportion of participants, we have set the recruitment target for *n* = 180 total, or *n* = 90 for each medication group. This will be facilitated by characteristics of design (few exclusion criteria, open-label allocation, outpatient data collection often coinciding with medication and counseling), prioritization of amount–and types of data collected. Information about the study will be distributed at clinical sites as well as outreach -, housing- and criminal justice facilities and the internet; these efforts are anticipated to attract the initial *n* = 30 patients whose presence in the larger community of opioid users will be sufficiently inform more potential participants to contact study staff for information.

#### Analysis sets

In addition to analyses of data from all patients who complete the 12-Week study, Intention-to-treat (ITT) analyses of efficacy endpoints will be conducted with all patient data. Separate analyses sets may be conducted on different analysis sets depending on publication requirements: e.g. a modified intention to treat (MITT) set of randomized patients who receive at least one dose of their assigned medication and complete at least one study follow-up; or a per-protocol dataset of patients who exhibit compliance with medication–and attend all four outpatient follow-ups. Imputation will be conducted using the previous valid assessment (last observation carried forward) at the last attended study follow-up or (if no follow-up took place) from the inclusion dataset; imputation using data modeling may be considered pending satisfactory similarity to collected data. The inclusion dataset may be corrected for days spent in a controlled environment (e.g. on-site detoxification unit) in order to convey the most accurate picture of pre-study functioning. For analyses beyond the 84-day randomized period, imputation (e.g. ITT, MITT) will only be conducted if considered relevant to the research question.

#### Dissemination of results

Scientific dissemination will occur under ICMJE guidelines for authorship and publication. Dissemination of results to a broader audience will be conducted via the University of Oslo's - and the research group’s websites, general news media in Norway and internationally.

## Discussion

This study has the potential to become the first comparison between extended-release naltrexone and daily buprenorphine-naloxone for opioid dependence. Such comparisons are currently lacking from the knowledge base [47]. In addition, longer-term data on the performance of XR-NTX in opioid dependent patients is central in evaluating the clinical feasibility of XR-NTX in this high-risk, chronically relapsing patient group. Registry linkage data provides an option to study the clinical trajectories of participants before, during and after the active follow-up phase, providing opportunities to address long-term recovery outcomes as well as concerns of increased overdose risk upon termination of naltrexone medication.

Parts of the original protocol have not been possible to implement: One site investigator has passed away prematurely without leaving a competent successor, leading to the premature closing of one site. A policy change greatly reduced the number of patients imprisoned for possession of minor quantities of drugs, meaning it has not been feasible to recruit a large proportion of participants from the criminal justice system. In order to maintain recruitment targets using only clinical sites, the inclusion phase of the study has had to be extended about by 25 % compared to the original schedule. As the clinical population of opioid users has a lower incidence of lethal overdose than opioid users discharged from criminal justice settings, overall mortality figures are expected to decrease below what is considered a meaningful basis for statistical estimation. Despite these implementation difficulties, we consider the protocol to retain its intended value as a ‘first of a kind’ comparison.
